# Early rehabilitation relieves diaphragm dysfunction induced by prolonged mechanical ventilation: a randomised control study

**DOI:** 10.1186/s12890-021-01461-2

**Published:** 2021-03-29

**Authors:** Zehua Dong, Ying Liu, Yubiao Gai, Pingping Meng, Hui Lin, Yuxiao Zhao, Jinyan Xing

**Affiliations:** 1 Department of Critical Care Medicine, The Affiliated Hospital of Qingdao University, No. 16, Jiangsu Road, Qingdao, 266000 Shandong China; 2 Department of Rehabilitation Medicine, The Affiliated Hospital of Qingdao University, Qingdao, 266000 Shandong China

**Keywords:** Early rehabilitation, Diaphragm ultrasound, Mechanical ventilation, Diaphragm dysfunction, Diaphragm thickening fraction

## Abstract

**Background:**

Prolonged mechanical ventilation (MV) induces diaphragm dysfunction in patients in the intensive care units (ICUs). Our study aimed to explore the therapeutic efficacy of early rehabilitation therapy in patients with prolonged MV in the ICU.

**Methods:**

Eighty eligible patients who underwent MV for > 72 h in the ICU from June 2019 to March 2020 were enrolled in this prospective randomised controlled trial. The patients were randomly divided into a rehabilitation group (n = 39) and a control group (n = 41). Rehabilitation therapy included six levels of rehabilitation exercises. Diaphragm function was determined using ultrasound (US).

**Results:**

Diaphragmatic excursion (DE) and diaphragm thickening fraction (DTF) were significantly decreased in all patients in both groups after prolonged MV (p < 0.001). The rehabilitation group had significantly higher DTF (p = 0.008) and a smaller decrease in DTF (p = 0.026) than the control group after 3 days of rehabilitation training. The ventilator duration and intubation duration were significantly shorter in the rehabilitation group than in the control group (p = 0.045 and p = 0.037, respectively). There were no significant differences in the duration of ICU stay, proportion of patients undergoing tracheotomy, and proportion of recovered patients between the two groups.

**Conclusions:**

Early rehabilitation is feasible and beneficial to ameliorate diaphragm dysfunction induced by prolonged MV and advance withdrawal from the ventilator and extubation in patients with MV. Diaphragm US is suggested for mechanically ventilated patients in the ICU.

*Trial registration* Chinese Clinical Trial Registry, ID: ChiCTR1900024046, registered on 2019/06/23.

**Supplementary Information:**

The online version contains supplementary material available at 10.1186/s12890-021-01461-2.

## Background

Mechanical ventilation (MV) is a life support technique that is routinely used in the intensive care units (ICUs) for patients with life-threatening conditions [1]. Prolonged MV and failure of weaning from MV are two major risk factors for morbidity and mortality in ICU patients and incur high economic costs [2]. Despite the lifesaving roles of MV in these patients, frequent application of MV leads to the occurrence of infectious and non-infectious complications, such as ventilator-associated pneumonia, pulmonary infections, and atelectasis [3].

The diaphragm is the prima donna of the muscles involved in the respiratory system, and diaphragm function is crucial for successful weaning from MV [4]. MV often induces diaphragm inactivity as a result of excessive or low pressure support, concurrent of critical illness polyneuropathy, patient-ventilator asynchrony and disseminated infection. This can lead to diaphragm myofiber atrophy and impaired diaphragm contraction, known as ventilator-induced diaphragmatic dysfunction (VIDD) [5]. VIDD is an important contributor to difficult liberation from MV, longer ICU stay, and increased ICU mortality, and is a predictor of prolongation of MV [6]. Ultrasound (US) is a non-invasive tool for quantifying diaphragmatic excursion (DE) and thickness and for assessing the structure and function of the diaphragm [7, 8]. Decreased diaphragm thickness is indicative of diaphragm atrophy, and diaphragmatic thickening fraction (DTF) is reflective of diaphragmatic effort [9]. Accumulating evidence has shown that ultrasonographic detection of the diaphragm is feasible, reproducible, and highly reliable [10, 11]. Recently, US measurement of the diaphragm has been suggested to be valuable for predicting the optimal time for extubation in mechanically ventilated patients [12].

Early mobilisation in the ICU is beneficial to critically ill patients by reducing the incidence of ICU-acquired weakness, accelerating the functional recovery of patients, and promoting liberation from the ventilator [13]. Comprehensive early rehabilitation therapy has been demonstrated to be both safe and effective in improving consciousness of ICU patients, decreasing the incidence of complications, and reducing the duration of ICU stay and MV [14]. Previous studies have shown that early rehabilitation can improve the outcomes of mechanically ventilated patients who are admitted to the ICU after coronary artery bypass surgery [15, 16]. However, the impact of early rehabilitation therapy on diaphragm function in mechanically ventilated patients in the ICU has not been fully investigated.

We performed a prospective randomised study to explore the effect of early mobilisation on diaphragm function and outcomes in patients with prolonged MV. Diaphragm US was used to investigate the diaphragm function of patients. The results of our study will aid delineate the role of early rehabilitation therapy in the clinical management of mechanically ventilated patients in the ICU.

## Methods

### Patients

This prospective randomised controlled trial was conducted in accordance with CONSORT (Additional file 1) in a 16-bed general ICU of the Affiliated Hospital of Qingdao University from June 2019 to March 2020. The trial was approved by the Ethics Committee of the Affiliated Hospital of Qingdao University (No. QYFY WZLL 25526) and is registered in the Chinese Clinical Trial Registry (No. ChiCTR1900024046). Written informed consent was obtained from the patients or their relatives.

Patients who were eligible for inclusion were required to meet the following criteria: (1) Prolonged MV (> 72 h); (2) stable oxygen saturation, fraction of inspired oxygen ≤ 55%, and positive end expiratory pressure (PEEP) ≤ 8 cmH_2_O; (3) dose of dopamine < 10 μg/kg/min and dose of epinephrine < 0.4 μg/kg/min; (4) mean arterial pressure > 75 mmHg and urine output > 1 mL/kg/h; (5) good healing of the incision after surgery; (6) normal cognitive function; and (7) no history of chronic mental illness or chronic obstructive pulmonary disease.

The exclusion criteria were as follows: (1) Inability to perform physical activities; (2) long-term MV prior to admission; (3) neurological comorbidities involving muscles; (4) irreversible disorders with a 6-month mortality rate of > 50% according to Acute Physiology and Chronic Health Evaluation II (APACHEII); (5) unsound limbs; (6) administration of glucocorticoids (prednisone or other corticosteroid dose equivalents > 20 mg/day) for at least 20 days prior to admission; (7) cardiopulmonary resuscitation before admission to the ICU; (8) radiotherapy or chemotherapy within the previous 6 months; (9) presence of comorbidities, including acute myocarditis, deep venous thrombosis/embolism, and cerebrovascular accident; and (10) unstable fractures.

### Randomisation

Eighty patients who met the inclusion criteria were enrolled in this study. A computer-generated allocation order with a block size of 4 was used with patients allocated at a 1:1 ratio to an early rehabilitation group or a control group. Finally, 39 patients were assigned to the early rehabilitation group (treated with early rehabilitation therapy in the ICU) and 41 patients were assigned to the control group (treated with standard care).

### Rehabilitation therapy

The early rehabilitation program was designed in accordance with the physical and psychological conditions of the patients. Rehabilitation therapy consisted of six levels: level 0, turning over once every 2 h for unconscious patients with unstable vital signs; level 1–2, in addition to turning over, maintaining joint range of motion to prevent muscle atrophy, and placing normal limb position for conscious patients who could sit up for at least 20 min, 3 times a day; level 3, similar to level 2, but sitting on the edge of the bed for patients who could perform upper-limb anti-gravity training; level 4, similar to level 3, but standing up or sitting in a chair for at least 20 min a day for patients who could perform lower-limb anti-gravity training; and level 5, patients actively moved from the bed and walked bedside. Level 1–5 exercises were suitable for conscious patients. Generally, level 1–3 exercises were selected for patients with tracheal intubation, while level 3–5 exercises were selected for patients with tracheotomy. For level 0 exercises, the patients were placed in a supine or orthopnoea position. The connecting wires of the electrocardiogram (ECG) monitor were separated, the drainage tubes were clamped, and all pipes were placed on the right side of the bed. Enteral nutrition or constraints were temporarily stopped. For patients using a ventilator, the condensate water in the ventilator pipe was dumped to prevent airway infection because of the backflow of water during turning over. The connection between the monitoring lead wire and the ventilator pipe was loosened appropriately to prevent pulling the patient. Two nurses stood on the same side of the bed, one holding the patient's shoulders and waist, and the other holding the patient's buttocks and popliteal fossa. The nurses simultaneously lifted the patient to the proximal side, before gently turning the patient to the opposite side. The patients were given percussion on the back, except for those who were contraindicated for percussion. A soft pillow was placed on the back, chest, and between the knees according to the requirements of the lateral position. Enteral nutrition was continued, and the patient’s vital signs were monitored to comprehensively evaluate the effect of turning over.

Rehabilitation therapy was terminated when the following criteria were met: (1) mean arterial pressure < 65 mmHg or > 120 mmHg; systolic or diastolic blood pressure of patients with pre-existing renal diseases decreased by more than 10 mmHg after treatment; (2) heart rate < 50 beats/min or > 140 beats/min; (3) occurrence of new arrhythmia or taking > 1 μg/kg/min of norepinephrine to maintain blood pressure; (4) inhaled oxygen concentration of 60.0% accompanied by PaO_2_ < 70 mmHg; (5) PEEP > 8 cmH_2_O; (6) pulse blood oxygen saturation (SpO_2_) decreased by 10% or < 85%; (7) respiration rate > 35 breaths/min; (8) body temperature > 38 °C; and (9) after rehabilitation exercise, the patient’s condition deteriorated or the patient became unconscious as a result of issues such as new sepsis, gastrointestinal bleeding, and chest pain. Reassessment of the above conditions was necessary on the second day of rehabilitation. After 3 days of rehabilitation training, patients in the rehabilitation group continued to undergo rehabilitation exercises until successful weaning from MV.

### Ultrasound assessment of diaphragm activity and function

US measurement of the diaphragm was performed for all patients as previously described [17, 18]. One well-trained investigator (Ying L) performed the US measurements after 24 h of MV (at 1-day after MV) and 3 days of rehabilitation training (at 4-day after MV) using continuous positive airway pressure (CPAP) mode. The measurement was stopped immediately if the patients displayed any sign of discomfort or distress.

Diaphragm function and activity were evaluated using two diaphragmatic parameters, DE and DTF. DE reflects the amplitude of the movement of the diaphragm during the respiratory cycle. The broadband linear array probe was placed at the junction of the midclavicular line or the anterior axillary line and the lower edge of the costal arch. The liver or spleen was set as the diaphragm acoustic window. The probe was pointed to the head and back, and DE was displayed under M-mode (time-motion mode). Three subsequent measurements were averaged to taken as the final value. DTF shows varied thickness of the diaphragm at end-expiration and end-inspiration. The maximum and minimum values of each breathing cycle were taken as the end-inspiratory diaphragm thickness (DTei) and the end-expiratory diaphragm thickness (DTee), respectively. DTF was calculated by DTF = (DTei − DTee)/DTee × 100%. The values for 3 consecutive respiratory cycles were recorded and the average value was taken as the final value. All patients underwent diaphragm US in a semi-recumbent position (bed slope of 45°); a right anterior subcostal view was preferred.

### Statistical analysis

The sample size was calculated based on the following formula, with DE as the primary outcome:

$${\text{n}} = \left[ {2\upsigma ^{2} ({\text{Z}}_{{{\upalpha /2}}} + {\text{Z}}_{\upbeta } )^{2} } \right]/\left[ {(\upmu _{1} -\upmu _{2} )^{2} } \right].$$

where σ = 0.4; Z_α/2_ = 1.96 when α = 0.05; Z_β_ = 0.84 when β = 0.2. μ_1_ in the rehabilitation group was estimated to be 1.5, and μ_2_ in the control group was estimated to be 1.25. Therefore, 40 participants per group were needed. Considering a dropout rate of 10%, the minimal sample size of each group was calculated to be 44. The sample size was minimized to 80 after an interim analysis.

All data were analysed using the SPSS ver. 20.0 MacIntosh package. Normality distribution of all data was checked by Shapiro—Wilk normality test. For the data conform to the normality distribution, parametric test was used to calculate the difference; otherwise, nonparametric test was utilized. Parametric and nonparametric data of descriptive statistics are expressed as mean ± standard deviation and median (interquartile range), respectively. DE and DTF at different times in the same group were compared using the paired *t*-test. Differences between rehabilitation group and control group were compared by independent samples *t* test. Nonparametric tests (Wilcoxon rank-sum test) were employed to compare continuous data between the groups. Categorical variables were compared using Fisher’s exact test. The level of statistical significance was set at p < 0.05.

## Results

### Baseline characteristics of patients

Eighty patients who were admitted to the ICU during the study period and met the inclusion criteria were enrolled in the study. The patients were randomly dichotomized into a rehabilitation intervention group (n = 39) and a control group (n = 41). The baseline characteristics of the two groups are shown in Table 1. There were no statistically significant differences in age, BMI, APACHEII score, and sex between the patients in the rehabilitation and control groups (p < 0.05). Males accounted for more than half of these patients (rehabilitation intervention group, 64.10%; control group, 56.10%). There was no statistically significant difference in the mean BMI between the two groups (rehabilitation group, 23.18 ± 3.32 kg/m^2^; control group, 23.22 ± 3.67 kg/m^2^).

**Table 1:** Baseline characteristics of the enrolled patients in two groups (mean ± standard deviation)

	Age (years)	BMI (kg/m^2^)	APACHEII score	Gender (male,n/%)
Rehabilitation group (n = 39)	59.05 ± 17.61	23.18 ± 3.32	15.90 ± 6.01	25 (64.10%)
Control group (n = 41)	64.44 ± 14.72	23.22 ± 3.67	17.78 ± 8.40	23 (56.10%)
p-value	0.141	0.955	0.254	0.501

Differences between different groups were calculated by independent samples *t* test. BMI, body mass index; APACHE II, Acute Physiology and Chronic Health Evaluation II

### Rehabilitation therapy relieved prolonged MV-induced diaphragm dysfunction

After 1 day of MV, rehabilitation therapy was administered to the rehabilitation intervention group for 3 days, whereas only standard care was given to the control group. All 39 patients in the rehabilitation intervention group completed the rehabilitation exercises. One patient experienced SpO_2_ < 90% and was dropped out from the study. The SpO_2_ was improved to the normal range, and the vital signs were stable following bed rest. No accidental extubation or other adverse events were reported during the study period.

**Fig. 1:**
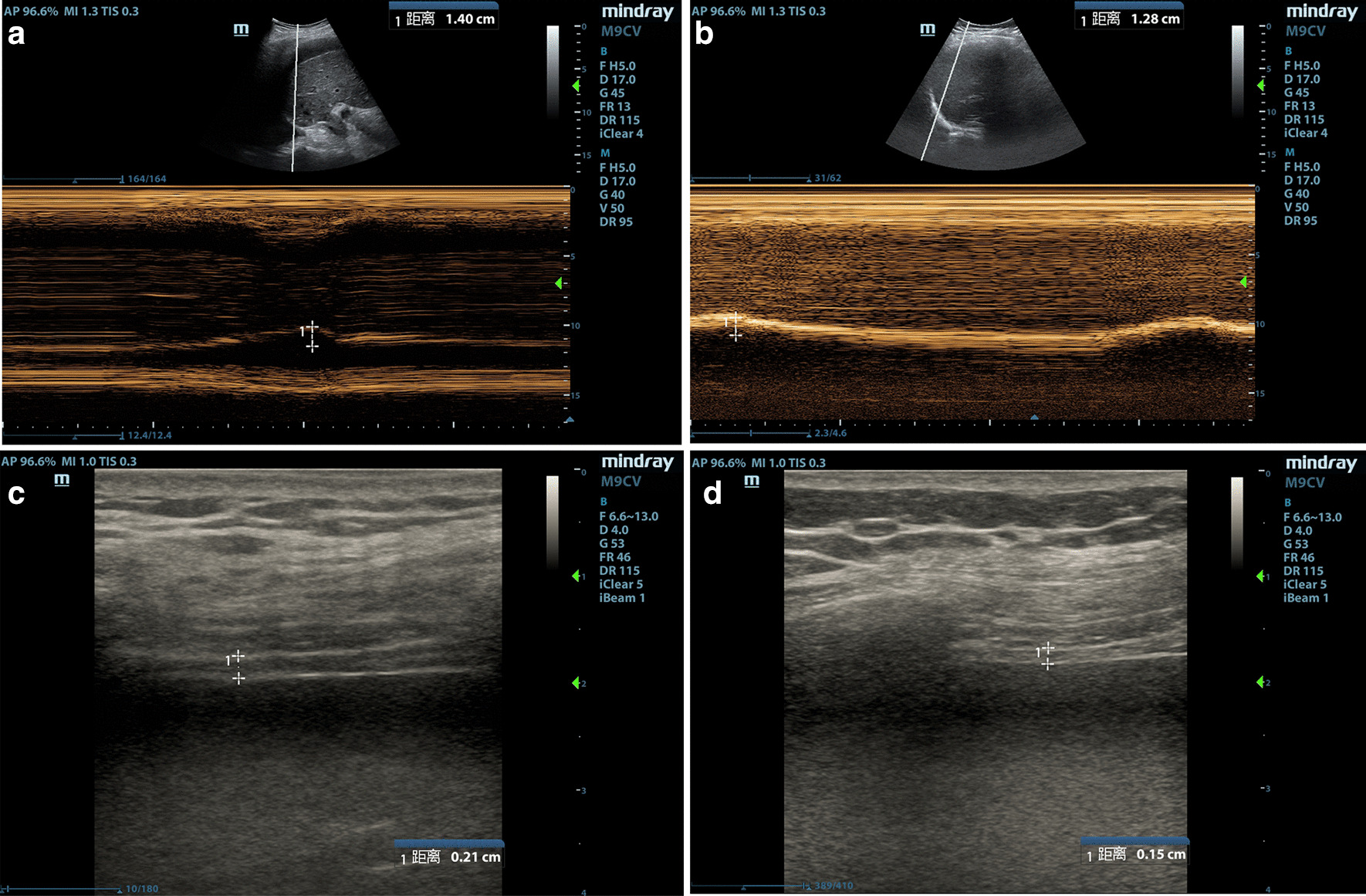
Example of ultrasonographic view before and after rehabilitation. **a** Diaphragmatic excursion (DE) was viewed during breathing in B-mode (upper) and M-mode (lower) before rehabilitation. **b** DE was viewed during breathing in B-mode (upper) and M-mode (lower) after rehabilitation. **c** DTF was viewed at the area of apposition before rehabilitation. **d** DTF was viewed at the area of apposition after rehabilitation

The two groups were assessed for DE and DTF using US at 1-day and 4-day MV. DE and DTF were significantly decreased in all patients at 4-day MV compared to those at 1-day MV (1.42 ± 0.53 vs. 1.30 ± 0.44, p < 0.001; 0.16 ± 0.06 *vs.* 0.13 ± 0.06, p < 0.001) (Fig. 1; Table 2). This suggests that prolonged MV impairs diaphragm function.

**Table 2:** DE and DTF values of all patients at 1-day MV and 4-day MV (mean ± standard deviation)

Time points	DE (cm)	DTF
1-day of MV	1.42 ± 0.53	0.16 ± 0.06
4-day of MV	1.30 ± 0.44	0.13 ± 0.06
p-value	< 0.001	< 0.001

Differences between different times were calculated by paired *t* test. MV, mechanical ventilation; DE, diaphragmatic excursion; DTF, diaphragm thickening fraction

**Fig. 2:**
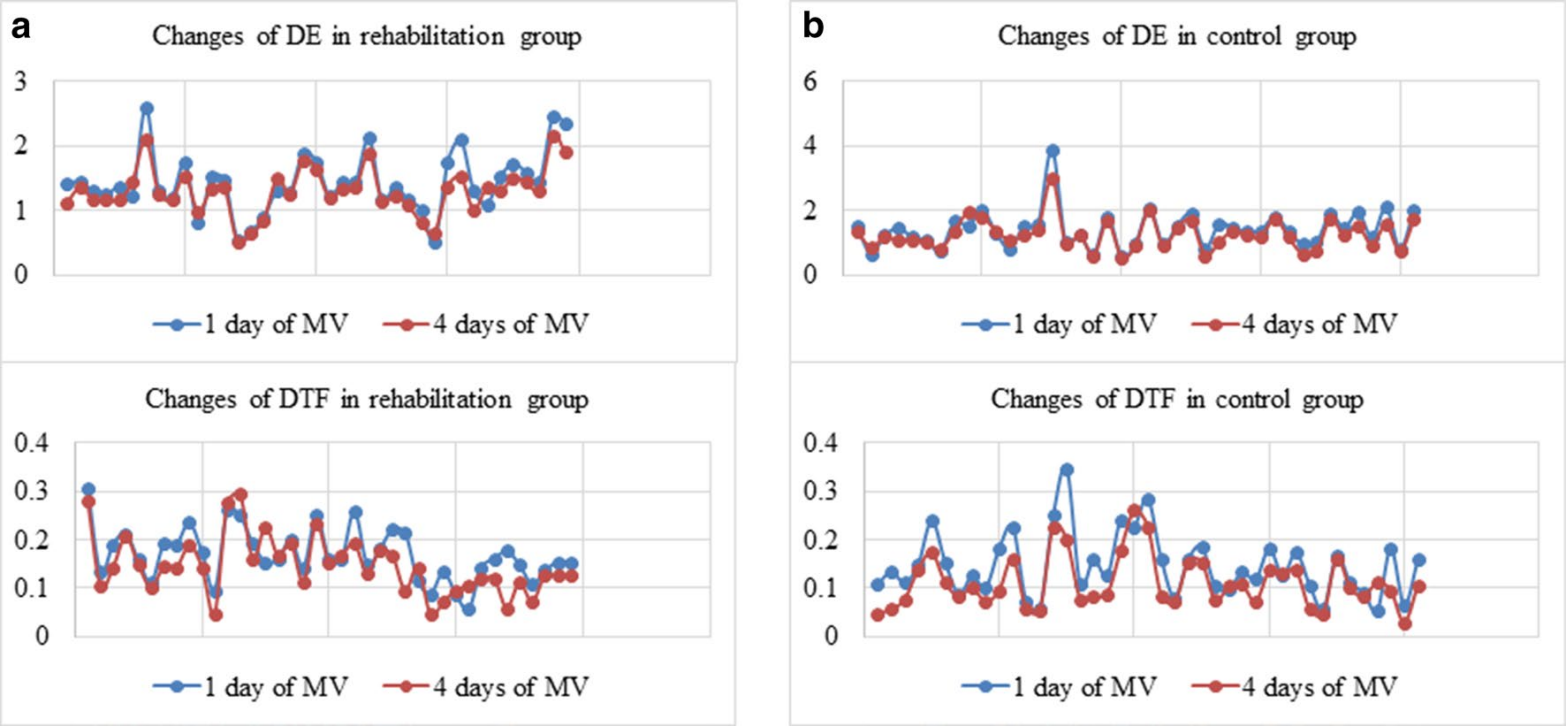
Changes of DE (upper panel) and DTF (lower panel) in rehabilitation group (**a**) and control group (**b**)

Before rehabilitation therapy, there were no significant differences in DE and DTF between both groups at 1-day MV (p-value > 0.05) (Table 3). At 4-day MV, the rehabilitation group had significantly decreased DTF compared to the control group (0.15 ± 0.06 g *vs.* 0.12 ± 0.05 g, p-value = 0.008) (Table 4). Changes of DE and DTF in rehabilitation group and control group were displayed in Fig. 2. Although a decline in DE was observed in the rehabilitation group relative to that in the control group, the decline was marginally significant (p = 0.541) (Table 4). We further found that the magnitude of the decrease in DTF before and after 3 days of rehabilitation therapy was obviously smaller in the rehabilitation group than in the control group (0.017 ± 0.030 *vs.* 0.034 ± 0.036, p = 0.026) (Table 4). These results reveal that early rehabilitation training can ameliorate diaphragm dysfunction caused by prolonged MV.

**Table 3:** Comparison of DE and DTF values in two groups at 1-day MV (mean ± standard deviation)

	DE (cm)	DTF
Rehabilitation group (n = 39)	1.43 ± 0.47	0.17 ± 0.05
Control group (n = 41)	1.41 ± 0.59	0.14 ± 0.07
t	0.189	1.740
Difference	0.022	0.023
95%CI for difference	− 0.215 to 0.260	− 0.003 to 0.051
p-value	0.851	0.086

Differences between different groups were calculated by independent samples *t* test. CI, confidence interval; DE, diaphragmatic excursion; DTF, diaphragm thickening fraction

**Table 4:** Comparison of DE and DTF values in two groups after 3 days of rehabilitation therapy (mean ± standard deviation)

	DE (cm)	DTF	Magnitude of decrease of DTF
Rehabilitation group (n = 39)	1.33 ± 0.39	0.15 ± 0.06	0.017 ± 0.030
Control group (n = 41)	1.27 ± 0.48	0.12 ± 0.05	0.034 ± 0.036
t	0.614	2.722	
Difference	0.060	0.035	
95% CI for difference	− 0.14 to 0.26	0.009 to 0.060	
p-value	0.541	0.008	0.026

Differences between different groups were calculated by independent samples *t* test. DE, diaphragmatic excursion; DTF, diaphragm thickening fraction

### Rehabilitation exercises improve the clinical outcomes of mechanically ventilated patients

The clinical outcomes of the rehabilitation intervention and control groups were compared (Table 5). The rehabilitation group had a significantly shorter duration of ventilator use (7.49 ± 2.59 days *vs.* 9.41 ± 5.32 days, p-value = 0.045) and a significantly shorter duration of intubation (8.31 ± 2.80 days *vs.* 10.37 ± 5.32 days, p = 0.037) than the control group (Table 5). The two groups were comparable in terms of duration of ICU stay, proportion of patients undergoing tracheotomy, and proportion of recovered patients (p-value > 0.05) (Table 5).

**Table 5:** Comparative analysis of clinical outcomes of two groups (mean ± standard deviation)

	Duration of ventilator (days)	Duration of intubation (days)	Length of ICU stay (days)	Tracheotomy (n/%)	Proportion of recovery (n/%)
Rehabilitation group (n = 39)	7.49 ± 2.59	8.31 ± 2.80	20.00 ± 12.67	7 (17.95)	34 (87.18)
Control group (n = 41)	9.41 ± 5.32	10.37 ± 5.32	21.12 ± 13.74	6 (14.63)	33 (80.49)
p-value	0.045	0.037	0.684	0.461	0.307

MV, mechanical ventilation

## Discussion

We found an obvious decline in DTF in our patients as a result of prolonged MV, which was significantly alleviated by 3 days of rehabilitation therapy. Early rehabilitation therapy shortened the duration of ventilator use and the duration of intubation in ICU patients with prolonged MV. Moreover, because of the strict safety criteria for rehabilitation training in the ICU setting, except for one minor transient event of desaturation, no other serious adverse events occurred in the patients who received rehabilitation therapy during the study period. Additionally, bedside monitoring of cardio-respiratory parameters showed the ability of patients with limited cardiac and respiratory reserve to perform exercises in bed (data not shown). Based on these results, we conclude that early rehabilitation training is feasible, safe, and beneficial for patients with prolonged MV in the ICU.

Critically ill patients frequently develop diaphragm weakness prior to ICU admission or during the ICU stay [19]. One identified critical risk factor for diaphragm weakness is the use of MV [20]. Diaphragm dysfunction is reported in over 60% of patients with MV and up to 80% of patients requiring prolonged MV with difficulty in weaning, and has a significant association with elevated ICU and hospital mortalities [8, 21]. Diaphragm dysfunction has a negative effect on the prognosis of mechanically ventilated patients by prolonging MV, delaying hospital discharge, and negatively affecting quality of life [22]. In the present study, we found diaphragmatic dysfunction in all enrolled patients as a consequence of prolonged MV, as evidenced by remarkable reductions in DE and DTF in all patients after 4-day MV.

Bed rest in conjunction with critical illness results in muscle wasting and weakness in the ICU [23]. Conversely, active mobilisation and rehabilitation in the ICU improves mobility status, increases muscle strength, and elongates the survival time of patients [24]. Growing evidence suggests that early mobilisation and rehabilitation is well tolerated and effective in accelerating the recovery of critically ill patients and should be implemented whenever indicated [25, 26]. Our study shows that early rehabilitation partly ameliorated the diaphragm dysfunction caused by prolonged MV, precipitated weaning from the ventilator, and shortened the length of intubation in patients with prolonged MV in the ICU, which is in concordance with the studies mentioned above. To the best of our knowledge, this is the first study to report the beneficial effect of early rehabilitation therapy on diaphragm dysfunction in patients with prolonged MV.

Ultrasonography is non-invasive and radiation-free and permits easy and accurate assessment of the diaphragm anatomy and function [27]. US equipment is ubiquitously present in ICU units, and diaphragm US can be conveniently performed at the bedside, without the need for additional efforts by patients [28]. Diaphragm US is reported to be a reliable indicator of inspiratory effort [29]. In light of these advantages, this study applied US to evaluate the diaphragm function of patients who underwent rehabilitation therapy. DE and DTF have been frequently proposed as an indicator of diaphragmatic contractile activity [30–33] or weaning predictor [34–36]. In this study, we found DTF, but not DE, was significantly reduced in the rehabilitation group compared to that in the control group. This indicates that DTF exhibits better performance than DE in evaluating the impact of rehabilitation therapy on diaphragm function. This result is in consistent with the study of Umbrello et al. which demonstrated that DTF is a reliable indicator of diaphragm contractile activity in critically ill patients [37].

Diaphragm dysfunction has been increasingly recognised as the primary reason for difficult weaning or weaning failure from MV, and measurement of diaphragm function using US has the potential to predict outcomes of weaning from MV [28, 38]. The current study found that patients who received rehabilitation therapy had significantly earlier withdrawal from the ventilator and earlier extubation than the control patients, implying that measurement of diaphragm function using DTF may be useful in predicting weaning and extubation in patients with prolonged MV in the ICU.

We acknowledge that the current study is limited by the insufficient sample size. Another limitation is the non-feasibility of diaphragm US in some cases, mainly due to non-comprehension or non-cooperation of the patients. Moreover, the enrolled patients with MV were not further stratified according to the extent of diaphragmatic dysfunction or diseases. Additionally, there was a lack of follow-up information on these patients after discharge. Further studies are warranted to verify the results of the current study.

## Conclusion

We conclude that early rehabilitation management is safe and effective in alleviating diaphragm dysfunction and could facilitate early withdrawal from the ventilator and early extubation in patients with prolonged MV in the ICU. US is a convenient and useful tool to study diaphragm function in mechanically ventilated patients. This study recommends early rehabilitation therapy for patients with prolonged MV whenever possible.

## Supplementary Information

**Additional file1.** The CONSORT checklist of this study.

## Data Availability

The datasets used or analysed during the current study are available from the corresponding author on reasonable request.

## Abbreviations

MV: Mechanical ventilation
ICU: Intensive care unit
DE: Diaphragmatic excursion
DTF: Diaphragm thickening fraction
VIDD: Ventilator-induced diaphragmatic dysfunction
CPAP: Continuous positive airway pressure
